# Lipidomics Reveals the Therapeutic Effects of EtOAc Extract of *Orthosiphon stamineus* Benth. on Nephrolithiasis

**DOI:** 10.3389/fphar.2020.01299

**Published:** 2020-08-21

**Authors:** Yufan Chao, Songyan Gao, Na Li, Hongxia Zhao, Yong Qian, Haihong Zha, Wei Chen, Xin Dong

**Affiliations:** ^1^School of Medicine, Shanghai University, Shanghai, China; ^2^Institute of Translational Medicine, Shanghai University, Shanghai, China; ^3^Shanghai Standard Technology Co., Ltd, Shanghai, China; ^4^SCIEX, Analytical Instrument Trading Co., Ltd, Shanghai, China; ^5^Department of Nephrology, Shanghai Changhai Hospital, Shanghai, China

**Keywords:** EtOAc extract of *Orthosiphon stamineus* Benth., nephrolithiasis, lipidomics, glycerophospholipids metabolism, inflammation, oxidative stress

## Abstract

**Background:**

Nephrolithiasis is a systemic metabolic disease with a high prevalence worldwide and is closely related to lipid-mediated oxidative stress and inflammation. *Orthosiphon stamineus* Benth. (OS) is a traditional medicinal herb mainly containing flavonoids, caffeic acid derivatives, and terpenoids, which has the effect of treating urinary stones. However, the active ingredients of OS for the treatment of kidney stones and their regulatory mechanisms remain unknown. As a powerful antioxidant, flavonoids from herbs can mitigate calcium oxalate stone formation by scavenging radical. Thus, this work focused on EtOAc extract of OS (EEOS, mainly flavonoids) and aimed to reveal the potential intrinsic mechanism of EEOS in the treatment of kidney stones disease.

**Methods:**

Firstly, 75% ethanol extract of OS was further extracted with EtOAc to obtain EtOAc extract containing 88.82% flavonoids. Secondly, the extract was subjected to component analysis and used in animal experiments. Then, an untargeted lipidomics based on ultrahigh performance liquid chromatography coupled with TripleTOF 5600 mass spectrometer (UPLC-QTOF-MS) was performed to test the lipid changes of kidneys in the control group, model group and EEOS treatment groups. Finally, multivariate statistical analysis was used to identify differences between the lipid profiles of mice in the model group and the EEOS group.

**Results:**

Fifty-one lipid metabolites were significantly different between the mice in the model group and the EEOS intervention group, including glycerophosphocholines, glycerophosphoethanolamines, glycerophosphoinositols, and glycerophosphoglycerols. And the composition of glycerophospholipids-esterified ω-3 polyunsaturated fatty acids and glycerophospholipid subclasses in the kidneys of the EEOS group significantly changed compared to model group.

**Conclusions:**

The EEOS can inhibit the stones formation by improving oxidative stress and inflammation mediated by glycerophospholipid metabolism. This study reveals the potential mechanism of EEOS for kidney stones treatment at the lipid molecule level, providing a new direction for further study of the efficacy of OS.

## Introduction

Nepholiathiasis, the third most common disease of the urinary tract, aggravates the economic burden of people and affects the life quality of patients because of the high prevalence and high recurrence rate (Zeng et al., 2017). At present, several significant therapeutic treatments has been achieved in nepholiathiasis, such as extracorporeal lithotripsy and percutaneous nephrolithotomy. However, these approaches have side effects such as hemorrhage, hypertension, tubular necrosis and subsequent renal sprain (Ackaert and Schröder, 1989), and cannot prevent the recurrence of stones. Therefore, it’s necessary to find new effective and less side effects treatment methods for kidney stones.

Herbal medicines has a significant effect on the treatment of kidney stones with few side effects. Recently, more and more researches on traditional herbal medicine in stone resistance have been made, such as the *Punica granatum* chloroform extract and *Punica grantum* methanol extract have an effective in decreasing the urolithiasis in male rats (Rathod et al., 2012) and the aqueous extract of *Taraxacum officinale* has an effective anti-crystallization activity (Yousefi Ghale-Salimi et al., 2018). *Orthosiphon stamineus* Benth. (OS, Barcode 00190087), also named Cat’s whiskers, is a medicinal plant of the family Lamiaceae and widely distributed in Southeast Asian and China. Published literature showed that the main compounds in OS are flavonoids, terpenes, and caffeic acid derivatives (Sumaryono et al., 1991; Tezuka et al., 2000). At present, researches on OS for the treatment of kidney diseases such as nephritis, kidney infection and cisplatin nephrotoxicity have been widely carried out (Hiromitsu et al., 2003; Pariyani et al., 2017; Sarshar et al., 2017), especially in the treatment of kidney stones. For instance, the 50% methanol extract of OS inhibits the calcium oxalate crystal growth (Dharmaraj et al., 2006) and aqueous extract of OS has protective effect in a calcium oxalate stone forming (Akanae et al., 2010). However, these studies usually focused on the total extracts of OS and lacked systematic explanations on the mechanism of extracts in the treatment of kidney stones.

Lipids are well known to be involve4d in occurrence and development of nephrolithiasis. Epidemiological studies have shown that the stone risk incidence increases in people with dyslipidemias (Armando Luis et al., 2008; Ho Won et al., 2014; Kirejczyk et al., 2015). The lipid content in the urine of patients with kidney stones is positively correlated to the extent of renal tubular damage and oxidative stress (Boonla et al., 2011). Emerging evidence indicates that oxidative stress and inflammatory responses are related to the formation of CaOx nephrolithiasis (Khan, 2014; Dominguez-Gutierrez et al., 2018; Sharma et al., 2019). And our previous study also demonstrated that the lipid-mediated oxidative stress and inflammation are closely related to the development of kidney stones (Chao et al., 2018). Oxidative stress induced cellular damage and inflammatory processes can promote aggregation and retention of CaOx crystals. Lipid peroxidation is an important component of oxidative stress, which may explain the increased risk of stones in people with abnormal lipid metabolism.

As a powerful antioxidant properties, flavonoids from herbs have exhibited radical-scavenging activity and have been proven to be effective for mitigating calcium oxalate stone formation (Byong Chang et al., 2006; Park et al., 2008), but it lacks systematic study on the anti-stones mechanism of flavonoids in OS. The 75% ethanol extract of OS was further extracted with EtOAc to obtain an EtOAc extract containing 85% flavonoids (Yu-Sen et al., 2012). Thus, this work focused on EtOAc extract of OS(EEOS) and aimed to reveal the potential intrinsic mechanism of EEOS in the treatment of kidney stones disease. An untargeted lipidomics based on UPLC-QTOF/MS platform was applied to study the differential lipids between glyoxylate-induced kidney stones mice and EEOS administration mice.

## Materials and Methods

### Chemicals and Reagents

Methanol, acetonitrile and isopropanol (HPLC grade) were purchased from Merck (Darmstadt, Germany). Formic acid was obtained from Fluka (Buchs, Switzerland). Ammonium formate and internal standard 1-heptadecenoyl-sn-glycero-3-phosphocholine were from Sigma-Aldrich (St. Louis, Missouri, USA). Chloroform (HPLC grade) was obtained from Sinopharm Chemical Reagent Co., Ltd. (Shanghai, China). Glyoxylic acid(50% in water) was purchased from TCI Development Co., Ltd. (Shanghai, China). Ultrapure water was prepared using a Milli-Q water purification system (Millipore Corp., Billerica, MA, USA).

### Preparation of EtOAc Extract From OS

Dried material of OS was obtained from Anguo (Hebei, China). The material was identified by Prof. Lian-na Sun (School of Pharmacy, Second Military Medical University, Shanghai, China). The extraction method is referred to Yu-Sen Zhong et al. (Yu-Sen et al., 2012). Briefly, the dry material of OS was boiled in 12 volumes of 75% ethanol (v/w) for 1h and the OS ethanol extracts were extracted with 3 volumes of petroleum ether, EtOAc and n-BuOH to obtain 3 fractions. And the component analysis of EEOS fraction was carried out by UPLC-Q-TOF/MS method and the detailed methodology of the experiment was shown in the supplementary materials section. As a intervention, the dry power of EEOS was prepared into a suspension of 24 mg/ml with 0.5% CMC-Na and stored at 4°C.

### Animal Experiments

Animal experiments followed the National Institutes of Health guide for the Care and Use of Laboratory Animals. Eighteen wild-type male C57BL/6 mice (7–8 weeks) were purchased from Shanghai SLAC laboratory Animal Co., Ltd.(Shanghai, China). Mice were housed in groups(control group, model group, EEOS group) of six per standard cage. The animal experiments lasted seven days and the mice were given drug at 8:30 am every day. Model group and EEOS group were injected with glyoxylate at a dose of 120 mg/kg/day and control group was injected with the same volume of saline. Two hours after injection of glyoxylate, the EEOS group was treated EEOS suspension by intragastric administration at a dose of 360 mg/kg/day, while the control group and model group were given 0.5% CMC-Na. In addition, Cystone was selected as the positive reference drug. The selection of the dose of EEOS administered and positive reference drug is described in the supplemental materials section.

### Samples Collection

On the seventh day of animal experiments, mice were anesthetized with 3% chloral hydrate and blood samples were collected by orbital puncture, and then the kidneys were collected. After clotting at 4°C for 2 h, the blood was centrifuged at 3,500 rpm for 15 min and the serum was saved for biochemical analysis. After removing the capsule and pelvis, three kidneys of each group were fixed in neutral 4% paraformaldehyde purchased from Servicebio company (Wuhan, China) and the others were immediately stored at −80°C.

### Histological and Biochemical Analysis

Kidneys were fixed in 4% paraformaldehyde and embedded in paraffin. Sections (3–4 μm) were prepared and dyed using the Von Kossa stain kit(calcium Stain). The principle of Von Kossa staining is metal replacement. Its main process includes: Section dewaxing to water, Silver nitrate reaction, Hematoxylin- Eosin staining, dehydration and sealing. Calcium deposition was estimated by observing stained plaques.

The contents of serum creatinine and blood urea nitrogen were measured using a Chemray 240 automatic biochemistry analyzer (Shenzhen, Guangdong, China). Six serum samples from each group were tested for serum creatinine and blood urea nitrogen.

### Samples Preparation and UPLC-QTOF/MS Analysis

Six kidney samples from each group for UPLC-QTOF/MS analysis. Kidney tissue was weighed and homogenized in methanol/chloroform/water (v/v/v,2:1:0.8) followed by the addition of a volume of chloroform and a volume of water to extract the lipids. After vortexing for 30 s, the mixed samples were placed at room temperature for 5 min and then centrifuged at 13,000 rpm for 10 min at 4°C and 200 μl of chloroform were transferred to an Eppendorf tube and evaporated under nitrogen. The dried extracts were reconstituted with 400 μl of an chloroform/methanol (1:1,v/v) solution and transferred to autosampler vials. To monitor system stability, a quality control (QC) sample was prepared by mixing the same volume of each sample.

UPLC-QTOF/MS analysis was performed on a CBM-20A/Alite HPLC system (Shimadzu, Japan) equipped with a TripleTOF 5600 mass spectrometer (AB Sciex, USA). Chromatographic separations were performed on a Waters XBridge™ BEH C18 column (2.1 mm × 100 mm, 2.5 μm, Waters, Milford, MA). The mobile phases consisted of 40:60 water: acetonitrile(A) and 9:10:81 acetonitrile: water: isopropanol (B), both containing 0.1% formic acid and 10mM ammonium formate. The flow rate was held constant at 0.3 ml/min and the injection volume was 1 μl. The gradient elution conditions were: 0min, 40% B; 3 min, 68% B; 5 min, 70% B; 7 min, 70% B; 12 min, 85% B; 15 min, 99% B; 19 min, 99% B; 19.1 min, 40% B. The entire chromatographic gradient time is 24 min and the column temperature was 45°C.

The mass spectrometer was operated in both positive and negative information-dependent acquisition (IDA) modes. The specific instrument parameters were as follow: the source temperature was 550°C; the ion source gas 1 and 2 were 60 psi and the curtain gas was 35 psi; the ion spray voltage floating was 5.5 kV in positive mode and 4.5 kV in negative mode. The accumulation time for full scan was 150 ms and the accumulation time for each IDA experiment was 55 ms. The mass range from m/z 100 to m/z 1,300 and the collision energy was set 45 eV. Eight spectra with an intensity threshold above 100 cps, isotope exclusion were set to 4 Da.

### Data Processing and Statistical Analysis

The raw data were converted into “Analysis Base File” (ABF) format files by ABF Converter 4.0.0 software. Then, all data were analyzed using MSDIAL 3.20 software, which can perform deconvolution, streamline criteria for peak identification and identify lipids. Finally, an alignment result, with a list of accurate mass, retention time, metabolite name, and corresponding intensities for all the detected peaks, was exported in.txt format. After the data were internal standard normalized, the resultant data matrices were introduced to the SIMCA-P 11.0 software for unsupervised principal component analysis (PCA) and partial least squares discriminate analysis (PLS-DA).

Statistical analysis was performed using SPSS21.0. The significant differences of three groups were tested by one-way ANOVA followed by a Turkey test for multiple comparisons. P < 0.05 and fold change value (FC) greater than 1.5 or less than 0.67 were considered statistically significant.

## Results

### Compounds Analysis in the EEOS

UPLC-QTOF/MS approach was applied to identify the compounds of EEOS fraction (**Figure S1**) and detailed method is listed in supplemental materials section. As shown in **Table S1**, twenty-two compounds, mainly flavonoids, also contain several phenolic acids and terpenes, were identified from the EEOS fraction. Qualitative analysis of the EtOAc extract was based on the precursor ion and product ions of the standards. Representative qualitative analysis results are shown in **Figures S2–S4**.

Using the rutin standard as a control, total flavonoids content of EEOS was determined by a colorimetric method, linear relationship between absorbance (A) and rutin concentration (C, μg/ml) by equation A= 0.0128C +0.0073(R^2^ = 0.999) (**Table S2**). The final results indicated that the total flavonoids content in EEOS was about 88.82% (**Table S3**). In addition, rosmarinic acid, eupatorin and salvigenin were selected as the indexes to test the reproducibility of the EEOS extraction. The relative standard derivation (RSD) values of the peak areas of each component were less than 5% in six sets of experiments (**Table S4**), indicating that the EEOS extraction is reproducible.

### Histological and Biochemical Analysis

**Figure 1:**
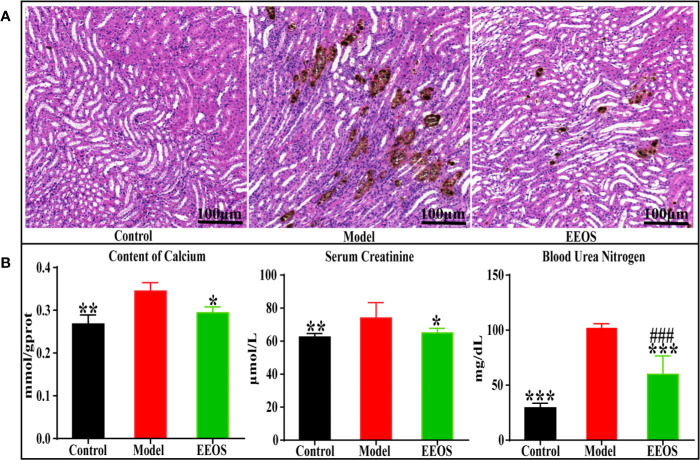
Tissue sections (×400) and biochemical indicator analysis. **(A)** Kidney slice stained with Von Kossa of the control group, model group and EtOAc extract of OS (EEOS) group. A large number of calcium spots were observed in the model group kidney sections and calcium spots in kidney stones mice are significantly reduced after taking EEOS; **(B)** the levels of Calcium, Serum creatinine and Blood urea nitrogen in the kidneys of three groups of mice. Data are expressed as mean ± SD. **^###^**P < 0.001 compared with the control group, *P < 0.05 compared with model group, **P < 0.01 compared with model group, ***P < 0.001 compared with model group.

To investigate whether EEOS could improve the development of stones in mice, histological analysis of renal sections using Von Kossa staining showed calcium spots in three groups mice (**Figure S5** and **Figure 1A**). Calcium spots was significantly reduced in renal sections by EEOS treatment compared with model group mice, which is consistent with the content reduction in calcium of mouse kidney homogenate after EEOS intervention (**Figure 1B**). Serum creatinine and blood urea nitrogen are routine indicators reflecting the state of renal function. Compared with model group, the serum creatinine and blood urea nitrogen levels are lower in the control group and EEOS group (**Figure 1B**). In addition, there were significant differences in blood urea nitrogen level between the EEOS group and the control group.

### Lipid Profiling and Multivariate Analysis of UPLC-QTOF/MS Data

**Figure 2:**
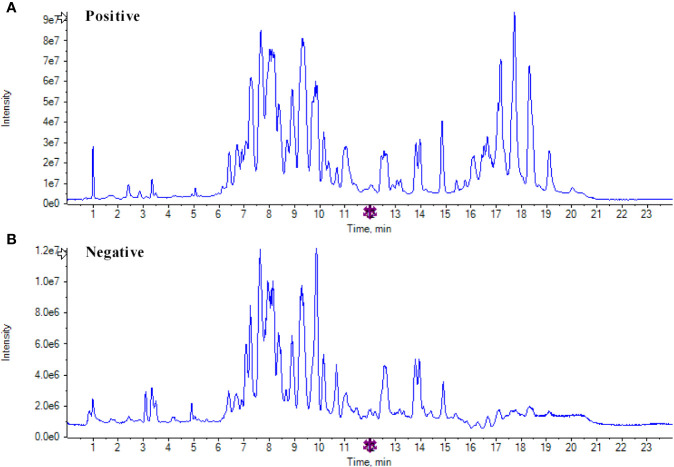
Representative total ion chromatograms for kidney samples. **(A)** Total ion chromatogram of quality control (QC) samples in positive mode; **(B)** Total ion chromatogram of QC samples in negative mode.

Lipidomics mainly recognizes important lipids in metabolic regulation, and reveals the mechanism of lipids in various life activities by comparing changes in lipid metabolism networks under different physiological conditions. By UPLC-MS/MS analysis, lipids in kidneys of three groups were detected in positive and negative ESI modes. Representative total ion chromatograms (TICs) for kidney samples from pooled QC sample are shown in **Figures 2A, B**. And, the coefficient of variation(CV) of QC sample was used to test reproducibility of the measured metabolites (**Figure S6**), and the ions with CV values greater than 20% in the positive and negative modes were approximately 10%, which indicated that the method has a well reproducibility and stability.

**Figure 3:**
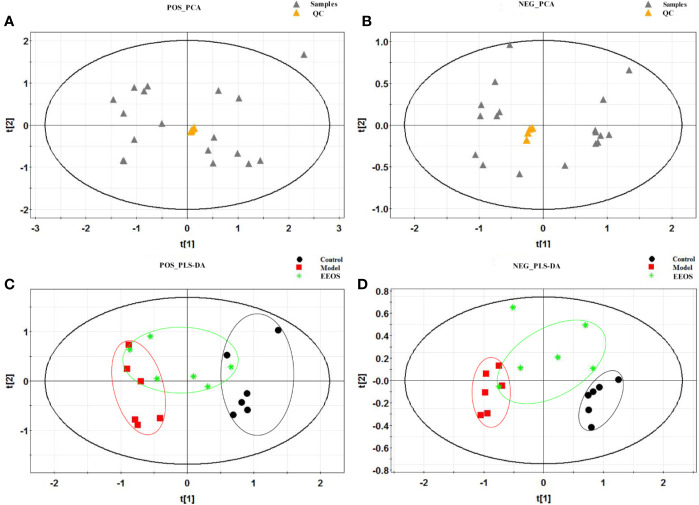
Multivariate statistical analysis score plots of three groups by in positive and negative ion modes. **(A)** Principal component analysis (PCA) score plot for all samples in positive mode, the quality control (QC) samples polymerized well; **(B)** PCA score plot for all samples in negative mode, the QC samples also polymerized well; **(C)** partial least squares discriminate analysis (PLS-DA) score plot in positive mode for control group, model group and EtOAc extract of OS (EEOS) group, the separation between the control group and the model group is obvious, and EEOS group has a tendency to rehabilitate from model group; **(D)** PLS-DA score plot in negative mode for control group, model group and EEOS group, there is good separation between control and model groups, and EEOS group has a tendency to recover to control group.

For multivariate statistical analysis, the unsupervised PCA model was firstly used to observe the discrete trend in the QC sample and judge the presence or absence of abnormal samples. It can be seen from the PCA score scatter plots (**Figures 3A, B**) that the QC sample is well polymerized, indicating that the instrument is in good operating condition. The supervised PLS-DA was applied to enhance the classification performance. As illustrated by PLS-DA score scatter plots (**Figures 3C, D**), model group was obviously separated from control group and the EEOS group had a significant tendency to closer to the control group than model group. In our established model for analysis, the cumulative R^2^X, R^2^Y and Q^2^ were above 0.4 (**Table S5**), indicating that the PCA and PLS-DA models were successfully.

### Lipids Identification and Analysis

**Figure 4:**
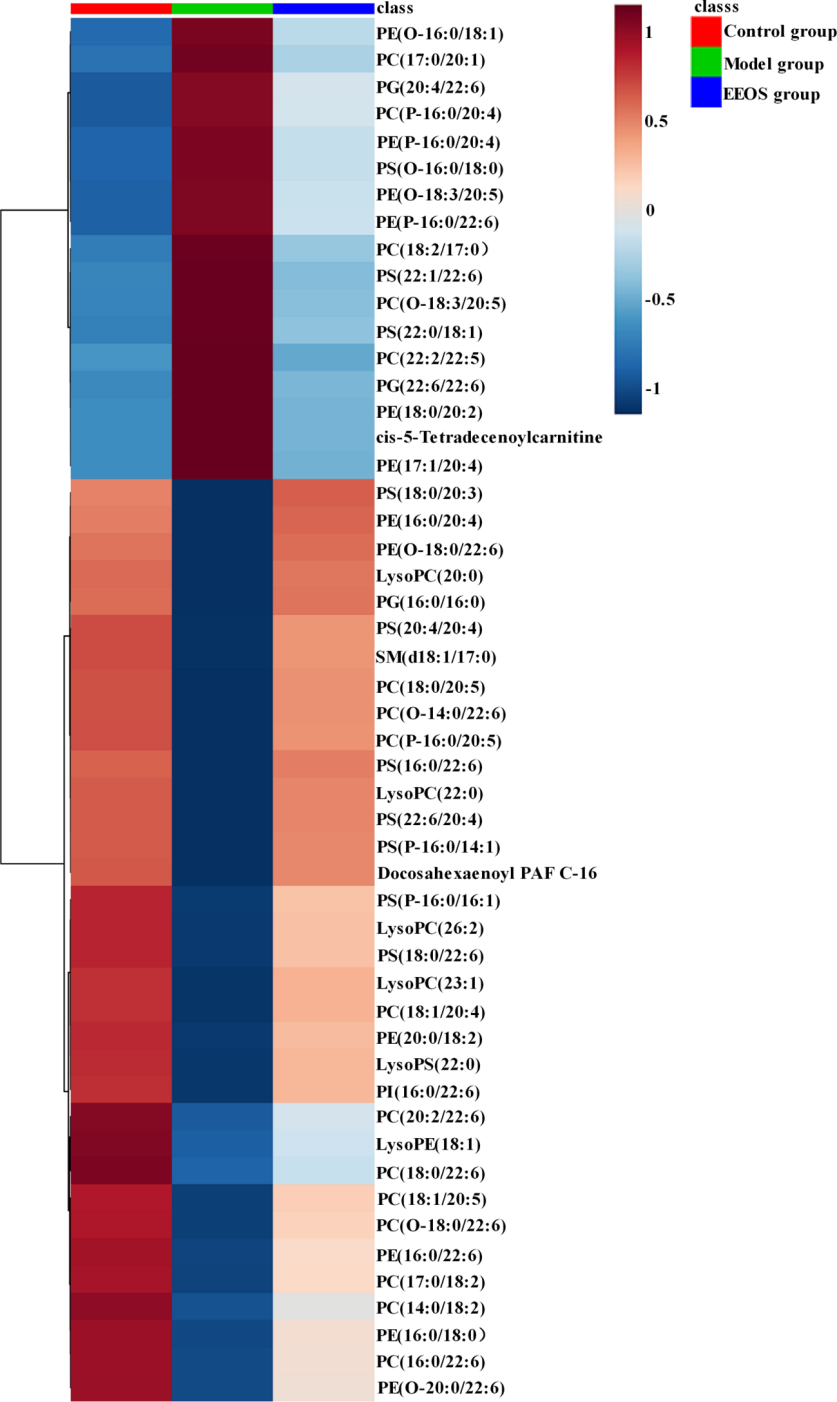
Hierarchical clustering analysis of three groups kidney samples. The lipid profiles of the control group and the model group were obviously different, while the lipid profiles of the control group and the EEOS group were similar. The depth of color represents the relative intensity of the lipid peak.

Based on the P value less than 0.05 and the FC value greater than 1.5 or less than 0.67, the differential ions with significant adjustment in the EEOS group were screened. According to the accurate mass-to-charge ratio and their MS/MS product ions (**Figure S7**), 51 differential lipids shown in **Table 1** were identified by database resources(MSDIAL, METLIN, and HMDB). These lipids mainly were glycerophospholipids including glycerophosphocholines(PC), glycerophosphoethanol- amines (PE), glycerophosphoserines (PS), glycerophosphoinositols (PI) and glycerophosphoglycerols (PG) (**Figure S8**). Cluster analysis with heat map to visualize the distribution of differential lipids in three groups of samples. As shown in **Figure 4**, the distribution of lipids in the model group was significantly different from that in the control group, but after treatment with EEOS, the lipids distribution of EEOS group was basically consistent with the control group, indicating that EEOS did interfere with the lipid metabolism of mice with stones.

**Table 1:** The list of differential lipids between EtOAc extract of OS (EEOS) group and model group.

No.	m/z(experimental value)	m/z(theoretical value)	Δppm	RT(min)	Adduct	Formula	Metabolite	VIP	FC*^a^*		Subclass
									Control/Model	EEOS/Model	
1	566.3808	566.3816	−1.41	2.81	[M+H]^+^	C_28_H_56_NO_8_P	LysoPC(20:0)*^c^*	0.17	0.11^##^	8.62^**^	Glycerophosphocholines
2	606.4131	606.4129	0.33	2.96	[M+H]^+^	C_31_H_60_NO_8_P	LysoPC(23:1)*^c^*	0.88	0.11^###^	7.26^***^	Glycerophosphocholines
3	594.4092	594.4129	−6.22	3.09	[M+H]^+^	C_30_H_60_NO_8_P	LysoPC(22:0)*^c^*	0.37	0.20^#^	4.67^*^	Glycerophosphocholines
4	646.4423	646.4442	−2.94	3.37	[M+H]^+^	C_34_H_64_NO_8_P	LysoPC(26:2)*^c^*	0.16	0.14^##^	5.28^*^	Glycerophosphocholines
5	806.567	806.5694	−2.98	5.17	[M+H]^+^	C_46_H_80_NO_8_P	PC(18:1/20:5)*^c^*	0.08	0.42^##^	1.88^*^	Glycerophosphocholines
6	852.5797	852.576	4.34	5.66	[M+FA-H]^-^	C_46_H_82_NO_8_P	PC(18:0/20:5)*^c^*	0.14	0.11^##^	7.90^*^	Glycerophosphocholines
7	808.5833	808.5851	−2.23	5.67	[M+H]^+^	C_46_H_82_NO_8_P	PC(18:1/20:4)*^b^*	0.13	0.24^##^	3.33^*^	Glycerophosphocholines
8	832.5522	832.5498	2.88	6.41	[M+FA-H]^-^	C_46_H_78_NO_7_P	PC(O-18:3/20:5)*^c^*	0.26	1.94^###^	0.60^**^	Glycerophosphocholines
9	808.5522	808.5498	2.97	6.73	[M+FA-H]^-^	C_44_H_78_NO_7_P	PC(O-14:0/22:6)*^c^*	0.09	0.59^###^	1.66^***^	Glycerophosphocholines
	764.5599	764.5589	1.31	6.74	[M+H]^+^	C_44_H_78_NO_7_P	PC(O-14:0/22:6)*^b^*	2.16	0.58^###^	1.62^***^	Glycerophosphocholines
10	786.5401	786.5408	−0.89	6.74	[M+Na]^+^	C_44_H_78_NO_7_P	PC(P-16:0/20:5)*^c^*	0.28	0.61^###^	1.55^**^	Glycerophosphocholines
11	850.5629	850.5604	2.94	7.26	[M+FA-H]^-^	C_46_H_80_NO_8_P	PC(16:0/22:6)*^b^*	0.09	0.50^###^	1.54^**^	Glycerophosphocholines
12	752.5222	752.5201	2.79	7.40	[M+Na]^+^	C_40_H_76_NO_8_P	PC(14:0/18:2)*^c^*	0.57	0.46^###^	1.56^*^	Glycerophosphocholines
13	810.5666	810.5654	1.48	7.54	[M+FA-H]^-^	C_44_H_80_NO_7_P	PC(P-16:0/20:4)*^b^*	0.14	4.27^###^	0.55^*^	Glycerophosphocholines
14	910.63	910.6296	0.44	8.13	[M+Na]^+^	C_52_H_90_NO_8_P	PC(22:2/22:5)*^c^*	0.32	2.29^#^	0.47^*^	Glycerophosphocholines
15	836.5835	836.5811	2.87	8.16	[M+FA-H]^-^	C_46_H_82_NO_7_P	Docosahexaenoyl PAF C-16*^b^*	0.17	0.57^##^	1.68^**^	Glycerophosphocholines
16	878.5943	878.5917	2.96	8.87	[M+FA-H]^-^	C_48_H_84_NO_8_P	PC(18:0/22:6)*^b^*	0.34	0.36^###^	1.66^*^	Glycerophosphocholines
17	820.6238	820.6215	2.80	9.96	[M+H]^+^	C_48_H_86_NO_7_P	PC(O-18:0/22:6)*^b^*	0.31	0.50^###^	1.64^*^	Glycerophosphocholines
18	858.5995	858.6007	−1.40	10.09	[M+H]^+^	C_50_H_84_NO_8_P	PC(20:2/22:6)*^b^*	0.14	0.44^###^	1.53^*^	Glycerophosphocholines
19	772.5871	772.5851	2.59	11.59	[M+H]^+^	C_43_H_82_NO_8_P	PC(18:2/17:0)*^c^*	0.22	1.91^###^	0.62^***^	Glycerophosphocholines
20	772.5858	772.5851	0.91	11.74	[M+H]^+^	C_43_H_82_NO_8_P	PC(17:0/18:2)*^c^*	0.50	0.44^###^	1.75^*^	Glycerophosphocholines
21	802.6339	802.632	2.37	14.02	[M+H]^+^	C_45_H_88_NO_8_P	PC(17:0/20:1)*^c^*	0.98	2.26^###^	0.60^**^	Glycerophosphocholines
22	480.3032	480.3085	−11.03	2.64	[M+H]^+^	C_23_H_46_NO_7_P	LysoPE(18:1)*^b^*	0.32	0.15^###^	3.17^*^	Glycerophosphoethanolamines
23	744.4974	744.4974	0.00	6.72	[M-H]^-^	C_43_H_72_NO_7_P	PE(O-18:3/20:5)*^b^*	0.59	3.90^###^	0.54^*^	Glycerophosphoethanolamines
24	746.5112	746.5095	2.28	6.74	[M+Na]^+^	C_41_H_74_NO_7_P	PE(P-16:0/20:4)*^b^*	1.17	3.81^###^	0.53^*^	Glycerophosphoethanolamines
25	762.5097	762.5079	2.36	7.06	[M-H]^-^	C_43_H_74_NO_8_P	PE(16:0/22:6)*^b^*	3.13	0.46^###^	1.68^*^	Glycerophosphoethanolamines
26	738.5108	738.5079	3.93	7.23	[M-H]^-^	C_41_H_74_NO_8_P	PE(16:0/20:4)*^b^*	0.68	0.63#	1.61^*^	Glycerophosphoethanolamines
27	750.5109	750.5079	4.00	7.35	[M-H]^-^	C_42_H_74_NO_8_P	PE(17:1/20:4)*^b^*	1.06	1.81#	0.60^*^	Glycerophosphoethanolamines
28	770.5093	770.5095	−0.26	7.65	[M+Na]^+^	C_43_H_74_NO_7_P	PE(P-16:0/22:6)*^b^*	1.99	2.37^###^	0.65^*^	Glycerophosphoethanolamines
29	776.5621	776.56	2.70	8.16	[M-H]^-^	C_45_H_80_NO_7_P	PE(O-18:0/22:6)*^b^*	1.18	0.62^##^	1.62^**^	Glycerophosphoethanolamines
30	828.5887	828.5878	1.09	9.07	[M+Na]^+^	C_47_H_84_NO_7_P	PE(O-20:0/22:6)*^c^*	3.26	0.39^###^	1.86^*^	Glycerophosphoethanolamines
31	720.5538	720.5538	0.00	9.53	[M+H]^+^	C_39_H_78_NO_8_P	PE(16:0/18:0)*^c^*	0.65	0.34^###^	2.08^*^	Glycerophosphoethanolamines
32	770.5737	770.5705	4.15	11.58	[M-H]^-^	C_43_H_82_NO_8_P	PE(18:0/20:2)*^c^*	0.55	2.23^###^	0.51^***^	Glycerophosphoethanolamines
33	770.5725	770.5705	2.60	11.72	[M-H]^-^	C_43_H_82_NO_8_P	PE(20:0/18:2)*^c^*	0.38	0.27^###^	2.94^*^	Glycerophosphoethanolamines
34	702.5461	702.5443	2.56	12.13	[M-H]^-^	C_39_H_78_NO_7_P	PE(O-16:0/18:1)*^b^*	0.67	1.99^###^	0.67^***^	Glycerophosphoethanolamines
35	881.521	881.5186	2.72	6.24	[M-H]^-^	C_47_H_79_O_13_P	PI(16:0/22:6)*^b^*	0.27	0.52^##^	1.68^*^	Glycerophosphoinositols
36	865.5061	865.5025	4.16	4.91	[M-H]^-^	C_50_H_75_O_10_P	PG(22:6/22:6)*^b^*	1.00	2.22^#^	0.52^*^	Glycerophosphoglycerols
37	841.5052	841.5025	3.21	5.07	[M-H]^-^	C_48_H_75_O_10_P	PG(20:4/22:6)*^b^*	0.17	2.90^###^	0.62^*^	Glycerophosphoglycerols
38	721.5042	721.5025	2.36	7.91	[M-H]^-^	C_38_H_75_O_10_P	PG(16:0/16:0)*^b^*	1.38	0.40^#^	2.48^*^	Glycerophosphoglycerols
39	596.3554	596.3558	-0.67	2.32	[M+H]^+^	C_28_H_54_NO_10_P	LysoPS(22:0)*^c^*	4.05	0.18^##^	4.28^*^	Glycerophosphoserines
40	690.4678	690.4704	−3.77	3.48	[M+H]^+^	C_36_H_68_NO_9_P	PS(P-16:0/14:1)*^c^*	2.12	0.24^###^	3.81^***^	Glycerophosphoserines
41	718.4997	718.5017	−2.78	4.25	[M+H]^+^	C_38_H_72_NO_9_P	PS(P-16:0/16:1)*^c^*	0.52	0.24^###^	3.23^**^	Glycerophosphoserines
42	912.5731	912.5725	0.66	4.93	[M+Na]^+^	C_50_H_84_NO_10_P	PS(22:1/22:6)*^c^*	0.34	2.92^##^	0.44^*^	Glycerophosphoserines
43	814.5608	814.5593	1.84	4.96	[M+H]^+^	C_44_H_80_NO_10_P	PS(18:0/20:3)*^c^*	3.48	0.25^##^	4.18^**^	Glycerophosphoserines
44	854.4989	854.4978	1.29	5.47	[M-H]^-^	C_48_H_74_NO_10_P	PS(22:6/20:4)*^b^*	0.50	0.52#	1.84^*^	Glycerophosphoserines
45	830.5016	830.4978	4.58	5.69	[M-H]^-^	C_46_H_74_NO_10_P	PS(20:4/20:4)*^c^*	0.33	0.49^##^	1.89^**^	Glycerophosphoserines
	832.5114	832.5123	−1.08	5.72	[M+H]^+^	C_46_H_74_NO_10_P	PS(20:4/20:4)*^c^*	0.63	0.49^##^	1.85^*^	Glycerophosphoserines
46	806.4977	806.4978	−0.12	5.87	[M-H]^-^	C_44_H_74_NO_10_P	PS(16:0/22:6)*^b^*	0.39	0.56^##^	1.74^**^	Glycerophosphoserines
47	836.5544	836.5436	12.91	6.21	[M+H]^+^	C_46_H_78_NO_10_P	PS(18:0/22:6)*^b^*	0.27	0.42^###^	1.94^**^	Glycerophosphoserines
48	750.5634	750.5643	−1.20	7.39	[M+H]^+^	C_40_H_80_NO_9_P	PS(O-16:0/18:0)*^c^*	0.29	4.14^###^	0.52^*^	Glycerophosphoserines
49	846.6235	846.6219	1.89	12.61	[M+H]^+^	C_46_H_88_NO_10_P	PS(22:0/18:1)*^b^*	0.33	2.05^##^	0.58^**^	Glycerophosphoserines
50	370.2911	370.2952	−11.07	3.53	[M+H]^+^	C_21_H_39_NO_4_	cis-5-Tetradecenoylcarnitine*^c^*	0.26	2.82^###^	0.42^***^	Fatty acyls
51	739.5708	739.5724	−2.16	11.5	[M+Na]^+^	C_40_H_81_N_2_O_6_P	SM(d18:1/17:0)*^c^*	0.31	0.31^##^	2.88^*^	Phosphosphingolipids

^#^P < 0.05, ^##^P < 0.01 and ^###^P < 0.001, compared with control group.

^*^P < 0.05, ^**^P < 0.01 and ^***^P < 0.001, compared with model group.

a FC The ratio of the mean of model group to the mean of control group and the ratio of the mean of EEOS group to the mean of model group.

b Lipids identified based on MS/MS product ions.

c Lipids identified based on m/z value.

#### Fatty Acids Composition in Glycerophospholipids

**Figure 5:**
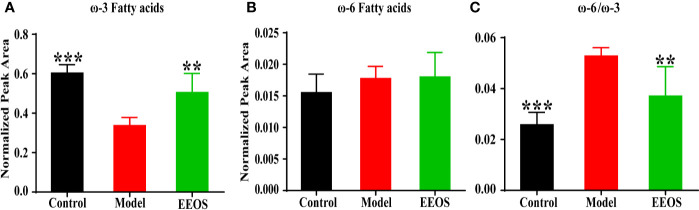
Changes in the composition of polyunsaturated fatty acids esterified by glycerophospholipids. **(A)** Differences in ω-3 fatty acids between the three groups. The ω-3 fatty acids in model group was significantly lower than that in control group and EEOS group; **(B)** ω-6 fatty acids no difference in ω-6 fatty acids between the three groups; **(C)** The ratio of ω-6 fatty acid chains to ω-3 fatty acid chains in glycerophospholipids was significantly increased in model groups compared with the control group and EEOS group. Data are expressed as mean ± SD. **P < 0.01, ***P < 0.001 vs. the model group.

Esterified fatty acids in glycerophospholipids can act as precursors of lipid mediators, and their compositional changes can affect the regulation of many physiological processes (Layé et al., 2018). Polyunsaturated fatty acids (PUFAs) refer to fatty acids having 18 or more carbon atoms and two or more double bonds in the chain, and can be classified into ω-3 fatty acids (linoleic acid and arachidonic acid) and ω-6 fatty acids according to the position of the double bond. The ω-3 fatty acids are mainly eicosapentaenoic acid and docosahexaenoic acid and the ω-6 series contains linoleic acid and arachidonic acid. Glycerophospolipids containing ω-3 fatty acids in the control and EEOS groups were significantly higher than the model group (**Figure 5A**). However, glycerophospolipids containing ω-6 fatty acids were not statistically different in the three groups (**Figure 5B**). The ω-6/ω-3 ratio was positively correlated with inflammation and oxidative stress (Lira et al., 2018). In the present study, the ω-6/ω-3 ratio (ratio of glycerophospholipids containing ω-6 fatty acids to glycerophospholipids containing ω-3 fatty acids) of model group dramatically increased than that in control group and EEOS group (**Figure 5C**).

#### Compositional Changes of Glycerophospholipids

**Figure 6:**
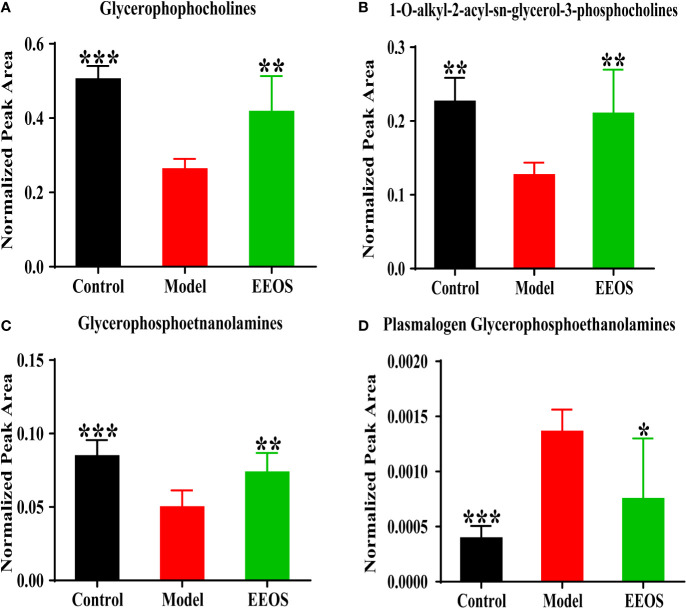
Analysis of glycerophosphocholines and glycerophosphoethanolamines in three groups. **(A)** The content of glycerophosphocholines in each group. The model group was significantly decreased compared with the control group and EtOAc extract of OS (EEOS) group; **(B)** analysis of 1-O-alkyl-2-acyl-sn-glycero-3-phosphocholines in three group. The model group was also significantly reduced compared with the control group and EEOS group; **(C)** The content of glycerophosphoethanolamines in each group. The model group was significantly decreased compared with the control group and EEOS group; **(D)** analysis of plasmalogen glycerophosphoethanolamines in three group. The model group was significantly increased compared with the control group and EEOS group. Data are expressed as mean ± SD. *P < 0.05, **P < 0.01, ***P < 0.001 vs. the model group.

Glycerophospholipids are the main components of cell membrane, which are necessary for cell survival and physiological function in organisms. Compared with model group, the total glycerophosphocholines increased significantly in EEOS group (**Figure 6A**), and 1-O-alkyl-2-acyl-sn-glycero-3-phosphocholines similar in structure to platelet activating factor(PAF) were also elevated (**Figure 6B**). Glycerophosphoethanolamines increased in control group and EEOS group compared to model group (**Figure 6C**), whereas plasmalogen glycerophosphoethanolamines(PE(P-)) have the opposite trend (**Figure 6D**).

**Figure 7:**
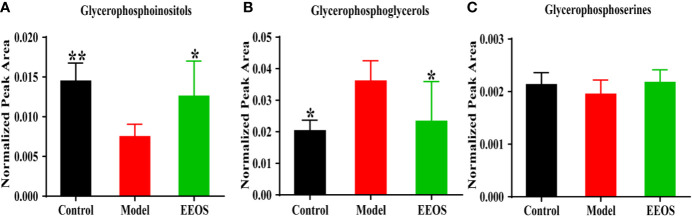
Analysis of acidic glycerophospholipids in three groups. **(A)** The content of glycerophosphoinositols in each group. The model group was significantly decreased compared with the control group and EtOAc extract of OS (EEOS) group; **(B)** Analysis of glycerophosphoglycerols in three group; **(C)** Glycerophosphoserines no difference between the three groups. Data are expressed as mean ± SD. *P < 0.05, **P < 0.01 vs. the model group.

The change of acidic glycerophospholipids was not consistent between the three groups. Glycerophosphoinositols obviously increased in control group and EEOS group compared to model group (**Figure 7A**). However, the glycerophosphoglycerols in control group and EEOS group are reduced compared with that in model group (**Figure 7B**). For glycerophosphoserines, there is no difference in the content of the three groups of samples (**Figure 7C**).

**Figure 8:**
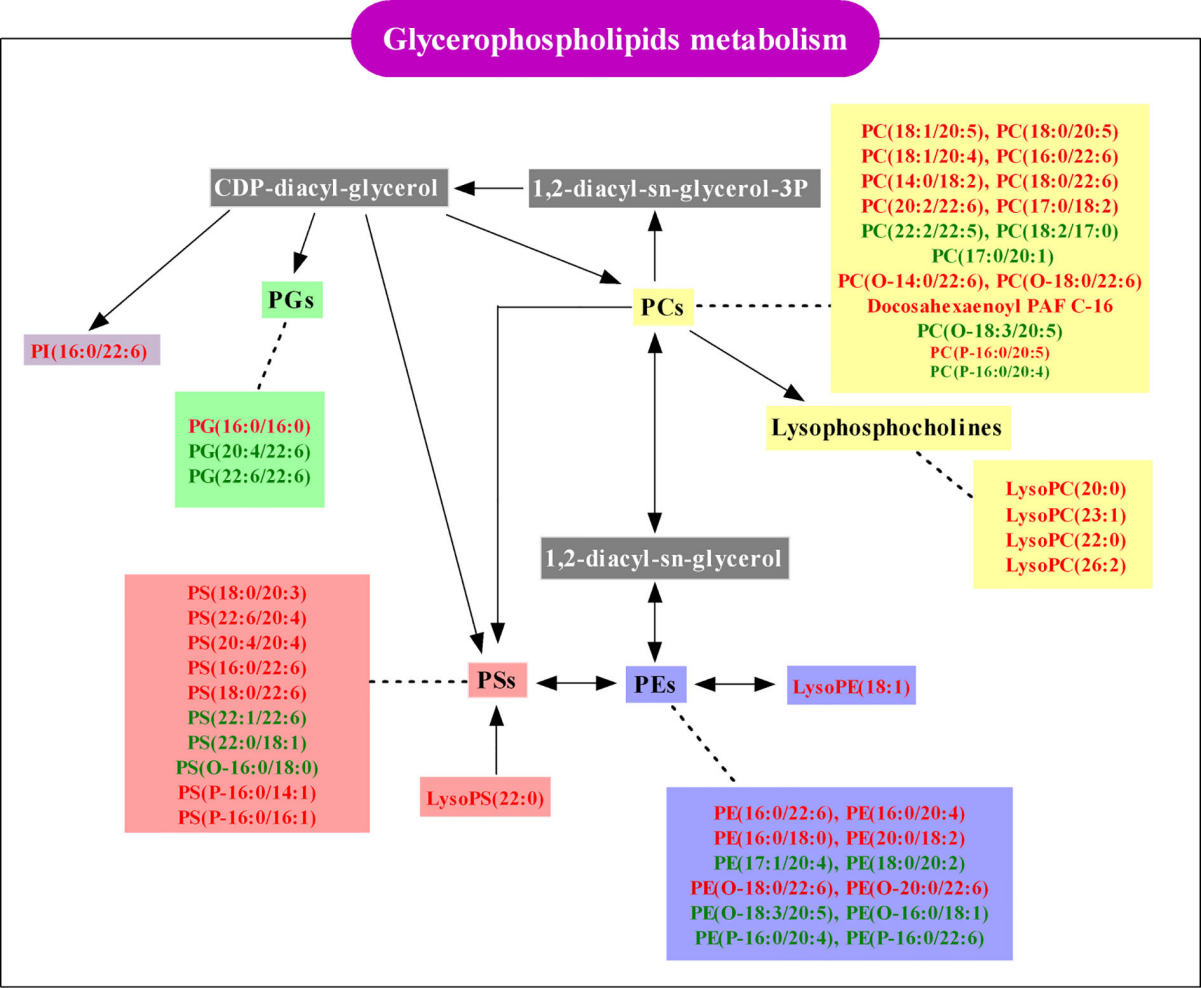
Schematic diagram of the glycerophospholipids metabolism pathway associated with EtOAc extract of OS (EEOS) fraction treatment of nephrolithiasis. Red-labeled lipids were elevated in the EEOS group and green-labeled lipids were reduced in the EEOS group compared to the model group. White-labeled lipids are undetected in this study.

Finally, 51 differential lipids were introduced into MetaboAnalyst 3.0 to further explore the metabolic pathway of EEOS against stone. It was found that glycerophospholipid metabolism may be a potential mechanism for EEOS to resist stone (**Figure S9**). Schematic diagram of the metabolic pathway related to EEOS fraction treatment of nephrolithiasis was shown in **Figure 8**.

## Discussion

Through the component analysis of the EEOS fraction, it was found to contain 88.82% flavonoids, which is consistent with that the flavonoids in EtOAc fraction of OS accounted for more than 85% (Yu-Sen et al., 2012). From histological and biochemical analysis, it can be see that EEOS fraction has a significant effect on reduction of kidney stones formation and has a certain recovery effect on the decline of renal function caused by stones. A total of 51 differential lipids with significant reversal trends were screened in the EEOS group, of which 49 lipids belong to glycerophospholipids, suggesting that the renal protective effects of EEOS fraction is closely related to the regulation of glycerophospholipid metabolism. And in previous studies, it was found that glycerophospholipids have potential relevance in the development of kidney stones disease (Chao et al., 2018). Therefore, this work mainly focuses on the anti-stone effect mechanism of EEOS from the perspective of glycerophospholipids metabolism.

### Changes of Fatty Acid Composition of Glycerophospholipids by EEOS Extract

Fatty acids metabolism is closely related to the formation of kidney stones (Takahiro et al., 2008; Hall et al., 2017; Bikulčienė et al., 2018). Esterified fatty acids in glycerophospholipids are potential precursor for lipid mediators, especially ω-3 fatty acids (Kang, 2012; Hishikawa et al., 2017). The concentration of ω-3 fatty acids in glycerophospholipids was obviously elevated in EEOS group mice than in model group, which is consistent with found that ω-3 fatty acids were significantly upper in normal people compared to patients with uronephrolithiasis (Bikulčienė et al., 2018), indicating that EEOS extract may reduce the occurrence of kidney stones by regulating the ω-3 fatty acids metabolism.

Peroxidase proliferator activates the receptor α (PPARα) is a master regulator of lipid metabolism, involved in inflammation and oxidative stress, and can be activated by ω-3 fatty acids. Haruya Takahashi et al. found lysophospholipids are increased on PPARα activation (Haruya et al., 2015). Meanwhile, the lysophospholipid content in the EEOS group was significantly higher than in the stone model group, which shown that the PPARα was more active in EEOS group mice than model group. PPARα antagonizes NF-κB-controlled proinflammatory mediator transcription by binding to NF-κB subunit p65 to form PPARα/NFκBp65 complexes (Jessica et al., 2011). In addition, ω-3 PUFAs can also reduce the expression of pro-inflammatory genes, such as the reduction of TNF-α expression by EPA (Adkins and Kelley, 2010). Although the glycerophospholipids esterified ω-6 fatty acids did not differ in the three groups in this study, there was a significant difference in the ratio of ω-6/ω-3. An unbalanced ω-6/ω-3 ratio is highly proinflammatory in terms of arachidonic acid metabolism and IL-1β production contributes to the prevalence of atherosclerosis and obesity (Simopoulos, 2008), which may be a potential factor for increased risk of stones in atherosclerosis patients and obese people.

Oxidative stress is caused by an imbalance between oxidant and antioxidant. Due to the instability of the double bond, PUFAs in glyperophospholipdis were easily oxidized under oxidative stress. The content of PUFAs in the EEOS group was significantly higher than that in the model group, indicating that the oxidative stress in the stone mice was alleviated after intervention with EEOS. Moreover, the vinyl double bond at the C-1 position of the glycerol backbone of the plasmalogens is preferentially oxidized under oxidative stress, preventing oxidation of polyunsaturated fatty acids and possibly mitigating cellular lipid peroxidation reactions (Lessig and Fuchs, 2009; Dean and Lodhi, 2018). In this study, the plasmalogen containing PUFAs in the model group was significantly higher than the control group, suggesting that the antioxidants in the stone mice were imbalanced and oxidative stress occurred. The plasmalogens in the EEOS group were also lower than in the model group, but the difference was not significant due to the large intra-difference in the EEOS group. Therefore, further research is needed on the effect of EEOS on the metabolism of plasmalogens. In addition, NF-κB upregulates the expression of pro-inflammatory cytokines and adhesion molecules to promote leukocyte adhesion, increase the release of oxygen free radicals and aggravate tissue damage (Holthe et al., 2004). In damaged cells and kidney tissues, crystals preferentially attach and aggregate into the nucleus, and the attached crystals can destroy the cells and form new tissue damage (Devarajan, 2018). From the aforementioned words, this study suggests that a potential mechanism of EEOS extracts in the treatment of stones may be through the regulation of fatty acid composition on glycerophospholipids to mediate oxidative stress and inflammation.

### Effects of EEOS on Subclasses of Glycerophospholipids

Glycerohospholipids account for about 70% of the total lipids in mammalian cells and are tightly related to the fluidity, flexibility, permeability and electrophysiological properties of biomembrane (Li et al., 2014). In addition to maintaining the integrity of cell membranes, PCs are also involved in the development of inflammatory reactions. PCs with 1-O-alkyl-2-acetyl-sn-glycerol-3-phosphocholine structure are called platelet activating factors(PAF) and are a class of lipid mediators with strong activity. In the kidney, PAF can induce intrarenal infiltration of inflammatory cells, activate mesangial cells and inflammatory cells to release a variety of inflammatory mediators; promote immune deposition in the kidneys and reduce glomerular filtration rate (Reznichenko and Korstanje, 2015). In present study, the acetyl group at sn-2 in PAF is replaced by ω-3 fatty acids, such as PC(O-14:0/22:6), PC(O-16:0/22:6), and PC(O-18:0/22:6), resulting in a decrease in the biological activity of PAF, which is beneficial to anti-inflammatory and prevent tissue damage.

PEs constituting 15%–25% of phospholipids and is very prominent in the energy metabolism of mitochondrion. Depletion of PE in mitochondria leads to dysfunctions in respiration and loss of mitochondrial DNA (Birner et al., 2003), resulting in insufficient energy metabolism in cells. PE in EEOS group was significantly higher than that in model group, indicating that EEOS may be related to mitochondrial productivity. In addition, plasmalogen glycerophosphoethanolamines(PE(P-)) are preferentially oxidized under oxidative stress (Lessig and Fuchs, 2009). In the research, the PE(P-16:0/20:4) and PE(P-16:0/22:6) in EEOS group were lower than that in model group, which suggested that the antioxidant activity of EEOS administration mice was enhanced to resist oxidative damage.

PI, PG and PS are acidic phospholipids and often recognized as second messengers. PI is a type of bioactive water soluble products of PLA2 and lysolipase and is an important precursor of secondary messenger phosphatidylinositol-3,4,5-triphosphate (PIP3) (Corda et al., 2009; Bohdanowicz and Grinstein, 2013). PIP3 can activate Ca^2+^ channels on cells and promote Ca^2+^ influx (Li et al., 2014) to regulate Ca^2+^ concentration intracellular and extracellular. In this study, the PI(16:0/22:6) elevated significantly in EEOS group compared to model group, which indicated that EEOS fraction may regulate the conversion of PI to PIP3, inhibiting the influx of Ca^2+^, thereby preventing cells from irreversible damage caused by Ca^2+^ overload. As an anionic glycerophospholipid, PG has an important role on lipid-protein topology and function (Frimmelová et al., 2016). However, Ca^2+^ can regulate ionic protein-lipid interactions by neutralizing the lipid negative charge, reducing the local concentration of acidic phospholipids (Li et al., 2014), which may be used to explain the decline of the PG in the EEOS group. PS is up to 10% of biological membrane and is the most abundant acid phospholipid (Platre and Jaillais, 2016). Although PS can promote cell proliferation and survival and mediate inflammatory responses by modulating cytokines (Maragno et al., 2015; Oyler-Yaniv et al., 2017), there were no significant differences in PS between the three groups in this study.

## Conclusion

This study focused on the therapeutic effect and potential mechanism of EEOS on glyoxylate-induced experimental kidney stones in mice. Lipidomics based on UPLC-QTOF-MS/MS platform was used to reveal the changes of lipids profile in kidneys of mice with kidney stones after intervention with EEOS, mainly the significant changes of glycerophospholipid metabolites containing polyunsaturated fatty acids. Significant changes in glycerophospholipids are not only a change in the composition of its subclasses such as glycerophospholipids, glycerophospholipids, and glycerophospholipids, but the esterified ω-3 fatty acid composition also undergoes significant changes. By analyzing differential metabolites, we found that the mechanism of anti-stone effect of EEOS is closely related to glycerolphospholipid-mediated oxidative stress and inflammatory response. Moreover, it is also closely related to Ca^2+^ metabolism involved in acidic phospholipids such as glycerophosphoglycerols and glycerophosphoinositols. In conclusion, this study reveals the mechanism of EEOS in the treatment of stone disease from the lipid molecular level, which provides a new direction for further study of the efficacy of *Orthosiphon stamineus* Benth.

## Data Availability Statement

All datasets presented in this study are included in the article/**Supplementary Material**.

## Ethics Statement

The animal study was reviewed and approved by: The experimental procedures were approved by the Ethical Committee for the Experimental Use of Animals at Second Military Medical University (Shanghai, China).

## Author Contributions

YC is the first author and performed all the experiments and wrote the manuscript. SG conceived the idea, designed the experimental plan and polished the whole manuscript. NL and HXZ helped the first author, prepared the animal experiments. YQ and HHZ provided technical assistance for samples test. WC and XD contributed toward study design, experimental setup, results supervision, and manuscript correction.

## Funding

This work was supported by Grants from the TCM Supported Project(15401900800) from the Science and Technology Commission of Shanghai Municipality.

## Conflict of Interest

YQ was employed by the company Shanghai Standard Technology Co., Ltd(Shanghai, China). HZ was employed by the company SCIEX, Analytical Instrument Trading Co., Ltd (Shanghai, China).

The remaining authors declare that the research was conducted in the absence of any commercial or financial relationships that could be construed as a potential conflict of interest.

## Abbreviations

EEOS, EtOAc extract of *Orthosiphon stamineus*; FC, fold change value; OS, *Orthosiphon stamineus* Benth.; PAF, platelet activating factor; PC, glycerophosphocholine; PCA, principal component analysis; PE, glycerophosphoethanolamine; PI, glycerophosphoinositol; PG, glycerophosphoglycerol; PS, glycerophosphoserine; PUFA, polyunsaturated fatty acid; QC, quality control; PLS-DA, partial least squares discriminate analysis.
