# Supplementary material for: Lipidomics Reveals the Therapeutic Effects of EtOAc Extract of Orthosiphon stamineus Benth. on Nephrolithiasis
Source: Front Pharmacol. 2020 Aug 21;11:1299. doi: 10.3389/fphar.2020.01299 (PMC7472562; doi:10.3389/fphar.2020.01299)
Supplement: Supplementary file 1 [file DataSheet_1.docx]

Supplementary Material

**Components analysis of EtOAc extract of *Orthosiphon stamineus* Benth.**

We analyzed the composition of the dried EtOAc extract of *Orthosiphon stamineus*(EEOS). The specific method is as follows: UPLC-QTOF/MS analysis was performed on Agilent 1290 Infinity LC system (Agilent, USA) equipped with Sciex TripleTOF 4600 LC/MS(AB Sciex, USA). Chromatographic separations were performed at 30°C on an Agilent  Zorbax Eclipse Plus C18 column(2.1 mm × 50 mm, 1.8μm, Agilent, USA). The mobile phase consisted of 0.1 % formic acid (A) and pure ACN (B). The elution gradients were: 0-2 min, 11% A; 2-4 min, 11%-30% A; 4-8 min, 30%-60% A; 8-10 min, 60%-90% A; 10-12 min, 90%-96% A; 12-12.5 min, 96%-11% A; 12.5-15 min, 11% A. The flow rate was 0.3 mL/min and the injection volume was 1μL. UV detection wavelengths were 203, 254 and 280 nm.

The mass spectrometer was operated in both positive and negative ion modes. The detailed TOF mass parameters were shown in below: the ion source temperature was 500°C; the ion source gas 1 and 2 were 50 psi; the curtain gas was 35 psi; the ion spray voltage floating was 5 kV in positive mode and 4.5 kV in negative mode. The mass range from m/z 100 to m/z 1,500 and the collision energy was set 10 eV.

In total, twenty-two compounds were identified from the EEOS, mainly flavonoids, in addition to phenolic acids and terpenes (**Table S1**). Qualitative analysis of EtOAc extracts was performed using a standard comparison method based on the characteristics that the same compound has the similar retention time and product ion under the same chromatographic conditions. The components in the extract were determined by comparing the retention time and product ion information of the sample peak and the standard peak. Representative qualitative analysis results are shown in **Figure S2**, **Figure S3** and **Figure S4**.

Moreover, UV-visible spectrophotometry was applied to detect the total flavonoids in EEOS with using rutin as standard which purchased from Shanghai Yuanye Biotechnology Co., Ltd. The specific method is as follows: first, accurate weighed 10.05mg of rutin standard, placed in a 50mL volumetric flask, dissolved in absolute ethanol and diluted to the scale to obtain the reference solution; then, accurately measured the reference solution 1mL, 2mL, 3mL, 4mL and 5mL, respectively, placed in a 25mL volumetric flask, diluted to prepare a series of standard solutions of 8.04μg/mL, 16.08μg/mL, 24.12μg/mL, 32.16μg/mL and 40.20μg/mL; finally, according to UV-visible spectrophotometry, measured the absorbance of standard solutions at 500nm wavelength. Linear relationship between absorbance(A) and rutin concentration(C, μg/mL) by equation A= 0.3879C +0.0073( R^2^= 0.999) is shown in **Table S2**. According to the above standard solution method, the EEOS test solution was prepared and the absorbance was detected. Finally, the total flavonoid content in the EEOS was calculated according to the rutin standard curve(**Table S3**).

In addition, rosmarinic acid, eupatorin and salvigenin were selected as the indexes to test the reproducibility of the EEOS extraction. The relative standard derivation (RSD) values of the peak areas of each component were less than 5% in six sets of experiments(**Table S4**), indicating that the EEOS extraction is reproducible.

**Determination of the dose of EEOS**

Combined with several researches on the anti-stone effect of *Orthosiphon stamineus* Benth. to select the dose of EEOS([Akanae et al., 2010](#_ENREF_1);[Yu-Sen et al., 2012](#_ENREF_2)). Finally, we examined the anti-stone efficacy of the low dose 90 mg/kg/d (LEEOS), medium dose 180mg/kg/d (MEEOS), and high dose 360mg/kg/d (HEEOS) of EEOS. Cystone is well known for its beneficial effects on the kidney and is used as a positive control drug in our studies on the therapeutic effect of drugs for urolithiasis. Histological analysis (**Figure S5**) shown that the intrarenal calcium spots in the HEEOS group were significantly less than those in the LEEOS group and MEEOS group, indicating that the efficacy of HEEOS was significantly better than the LEEOS and MEEOS. The calcium spots in the HEEOS group were similar to those in the positive drug Cystone group, indicating that the effect of HEEOS in the treatment of renal stones was similar to that of Cystone. Therefore, we finally chose the dose of 360mg/kg/d for the study of the anti-stone effect of EEOS.

**Table S1.** The list of 22 compounds in EEOS fraction.

| **No.** | **RT**  **(min)** | **Adduct** | **m/z**  **(actual value)** | **m/z**  **(theoretical value)** | **Δppm** | **Formula** | **Compounds name** | **Classification** | **MS/MS spetra** |
| --- | --- | --- | --- | --- | --- | --- | --- | --- | --- |
| 1 | 4.19 | [M-H]^-^ | 521.1296 | 521.1301 | -0.9 | C_24_H_26_O_13_ | salviaflaside | Phenolic acids | 359,323,197,179,161,97 |
| 2 | 4.49 | [M-H]^-^ | 470.145 | 470.1457 | -1.4 | C_24_H_25_NO_9_ | unknown | NA | 470,308,290,246,135 |
| 3 | 4.64 | [M-H]^-^ | 359.0773 | 359.0772 | 0.2 | C_18_H_16_O_8_ | rosmarinic acid | Phenolic acids | 197,179,161,133,123,72 |
| 4 | 4.85 | [M-H]^-^ | 717.1465 | 717.1461 | -1.3 | C_36_H_30_O_16_ | lithospermate Y | Phenolic acids | 519,339,321,295 |
| 5 | 4.89 | [M-H]^-^ | 470.1461 | 470.1457 | 0.9 | C_24_H_25_NO_9_ | unknown | NA | 470,308,290,272,246,161,135 |
| 6 | 5.06 | [M+H]^+^ | 314.1391 | 314.1387 | 1.3 | C_18_H_19_NO_4_ | N-trans-feruloyltyramine | Alkaloids | 314,177,145,121,117,103,89,77 |
| 7 | 5.49 | [M+H]^+^ | 331.0819 | 331.0824 | 2 | C_17_H_14_O_7_ | 3,3'-Di-O-methylquercetin | Flavonoids | 331,316,288,273,245,168,133 |
| 8 | 5.9 | [M+H]^+^ | 359.1133 | 359.1125 | 2.1 | C_19_H_18_O_7_ | 5-hydroxy-6,7,3',4'-tetramethoxyflavone or isomers | Flavonoids | 344,329,315,298,255,153 |
| 9 | 6.09 | [M+H]^+^ | 329.1019 | 329.102 | -0.2 | C_18_H_16_O_6_ | salvigenin or isomers | Flavonoids | 314,296,268,108 |
| 10 | 6.6 | [M+H]^+^ | 373.1289 | 373.1282 | 1.9 | C_20_H_20_O_7_ | sinensetin or isomers | Flavonoids | 357,343,329,312,181,163,153 |
| 11 | 6.78 | [M+H]^+^ | 345.0974 | 345.0969 | 1.5 | C_18_H_16_O_7_ | eupatorin | Flavonoids | 345,330,312,297,284,269,  241,148,136 |
| 12 | 7.14 | [M+H]^+^ | 343.1178 | 343.1176 | 0.5 | C_19_H_18_O_6_ | 5,7,3',4'-tetramethoxyflavone or isomers | Flavonoids | 313,282,253,239,181,153,135 |
| 13 | 7.63 | [M+NH_4_]^+^ | 588.2806 | 588.2803 | 0.5 | C_31_H_38_O_10_ | orthosiphol M or isomers | Terpenes | 588,571,389,371,329,311,  293,283,215,197 |
| 14 | 7.84 | [M+Na]^+^ | 487.3439 | 487.3429 | 2.1 | C_30_H_48_O_5_ | tormentic acid or isomers | Terpenes | NA |
| 15 | 7.88 | [M+Na]^+^ | 715.2472 | 715.2725 | 2.4 | C_38_H_44_O_12_ | orthosiphol L or isomers | Terpenes | 715,655,593,533,471,411 |
| 16 | 8.05 | [M+H]^+^ | 445.2129 | 445.2122 | 1.6 | C_27_H_28_N_2_O_4_ | asperglaucide | Alkaloids | 385,341,324,252,224,194,177,  147,134,117,105,91 |
| 17 | 8.16 | [M+Na]^+^ | 715.247 | 715.2725 | 2.1 | C_38_H_44_O_12_ | orthosiphol L or isomers | Terpenes | 715,655,593,533,471 |
| 18 | 8.24 | [M+H]^+^ | 329.1028 | 329.102 | 2.5 | C_18_H_16_O_6_ | salvigenin or isomers | Flavonoids | 314,296,268,240,197,169,  152,136,108 |
| 19 | 8.6 | [M+Na]^+^ | 655.2535 | 655.2514 | 3.3 | C_36_H_40_O_10_ | orthosiphol N or isomers | Terpenes | 633,591,511,469,451,347,329,  311,293,283,265,215,197,187 |
| 20 | 8.86 | [M+Na]^+^ | 699.2802 | 699.2776 | 3.7 | C_38_H_44_O_11_ | orthosiphol A or isomers | Terpenes | 699,577,455,395 |
| 21 | 9.06 | [M+Na]^+^ | 699.2796 | 699.2776 | 2.9 | C_38_H_44_O_11_ | orthosiphol A or isomers | Terpenes | 699,577,455,395 |
| 22 | 9.21 | [M+Na]^+^ | 697.2642 | 697.2619 | 3.3 | C_38_H_42_O_11_ | orthosiphonone A or isomers | Terpenes | 697,637,393 |

NA: No clear attribution.

**Table S2.**  Linear relationship between absorbance(A) and rutin concentration(C, μg/mL).

| **C(**μg/mL**)** | **A1** | **A2** | **A3** | **Mean A** | **Standard curve** |
| --- | --- | --- | --- | --- | --- |
| **8.04** | **0.1103** | **0.1111** | **0.1117** | **0.111** | **A=0.0128 C+0.0073**  **R^2^=0.999** |
| **16.08** | **0.2115** | **0.2101** | **0.21** | **0.2105** |  |
| **24.12** | **0.3156** | **0.3154** | **0.3141** | **0.315** |  |
| **32.16** | **0.4278** | **0.4276** | **0.4279** | **0.4278** |  |
| **40.2** | **0.5174** | **0.5171** | **0.5175** | **0.5173** |  |

Note: A1, A2, and A3 are the three absorbances in the measurement process, and finally the average absorbance (Mean A) is used to calculate the standard curve.

**Table S3.** Determination of total flavonoids content in EEOS.

| **Total C(**μg/mL**)** | **A1** | **A2** | **A3** | **Mean A** | **Measured C(**μg/mL**)** | **Content(%)** |
| --- | --- | --- | --- | --- | --- | --- |
|  |  |  |  |  |  | **Measured C/Total C** |
| **41.38** | **0.4812** | **0.4763** | **0.4797** | **0.4791** | **36.86** | **88.82** |
| **41.42** | **0.4771** | **0.4766** | **0.4767** | **0.4768** | **36.68** |  |

Note: Total C is the total concentration of the EEOS extract test solution; Measured C is the actually measured

concentration of flavonoids in the test solution.

**Table S4.** The reproducible results of the EEOS extraction.

| **Name** | **Wavelength** | **EEOS fractions( Peak Area)** | | | | | | **SD** | **Mean** | **RSD(%)** |
| --- | --- | --- | --- | --- | --- | --- | --- | --- | --- | --- |
|  |  | **EEOS-1** | **EEOS-2** | **EEOS-3** | **EEOS-4** | **EEOS-5** | **EEOS-6** |  |  |  |
| **rosmarini cacid** | **200nm** | **2143.00** | **2051.10** | **2093.02** | **2067.58** | **2124.39** | **2010.15** | **48.91** | **2081.54** | **2.35** |
| **eupatorin** | **230nm** | **117.85** | **118.35** | **112.48** | **109.70** | **113.40** | **111.70** | **3.47** | **113.91** | **3.04** |
| **salvigenin** | **328nm** | **53.41** | **53.16** | **50.35** | **49.36** | **50.05** | **49.64** | **1.81** | **51.00** | **3.54** |

**Table S5.** Summary of the parameters for assessing quality of PCA and PLS-DA model.

| **Model** | **Mode** | **No. Component** | **R^2^X** | **R^2^Y** | **Q^2^** |
| --- | --- | --- | --- | --- | --- |
| **PCA** | **Positive** | **4** | **0.898** | **—** | **0.75** |
|  | **Negative** | **4** | **0.885** | **—** | **0.712** |
| **PLS-DA** | **Positive** | **2** | **0.672** | **0.465** | **0.645** |
|  | **Negative** | **7** | **0.933** | **0.98** | **0.781** |

Note: "—" means null.

| **A** | 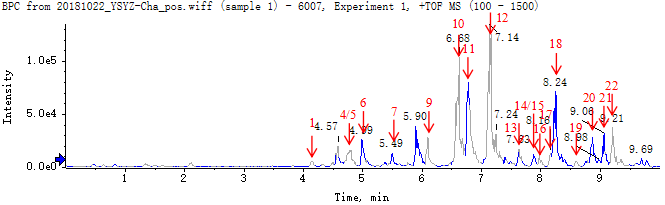 |
| --- | --- |
| **B** | 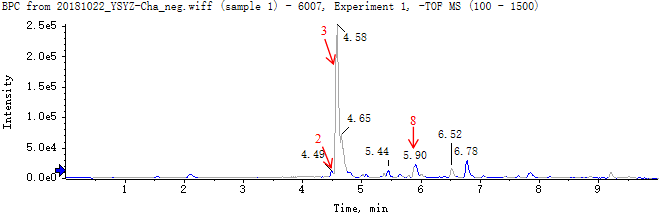 |

**Figure S1.** The compositional spectra of EEOS in ESI positive and negative ion modes. (A) The compositional spectrum of EEOS in ESI positive mode; (B) The compositional spectrum of EEOS in ESI negative mode.


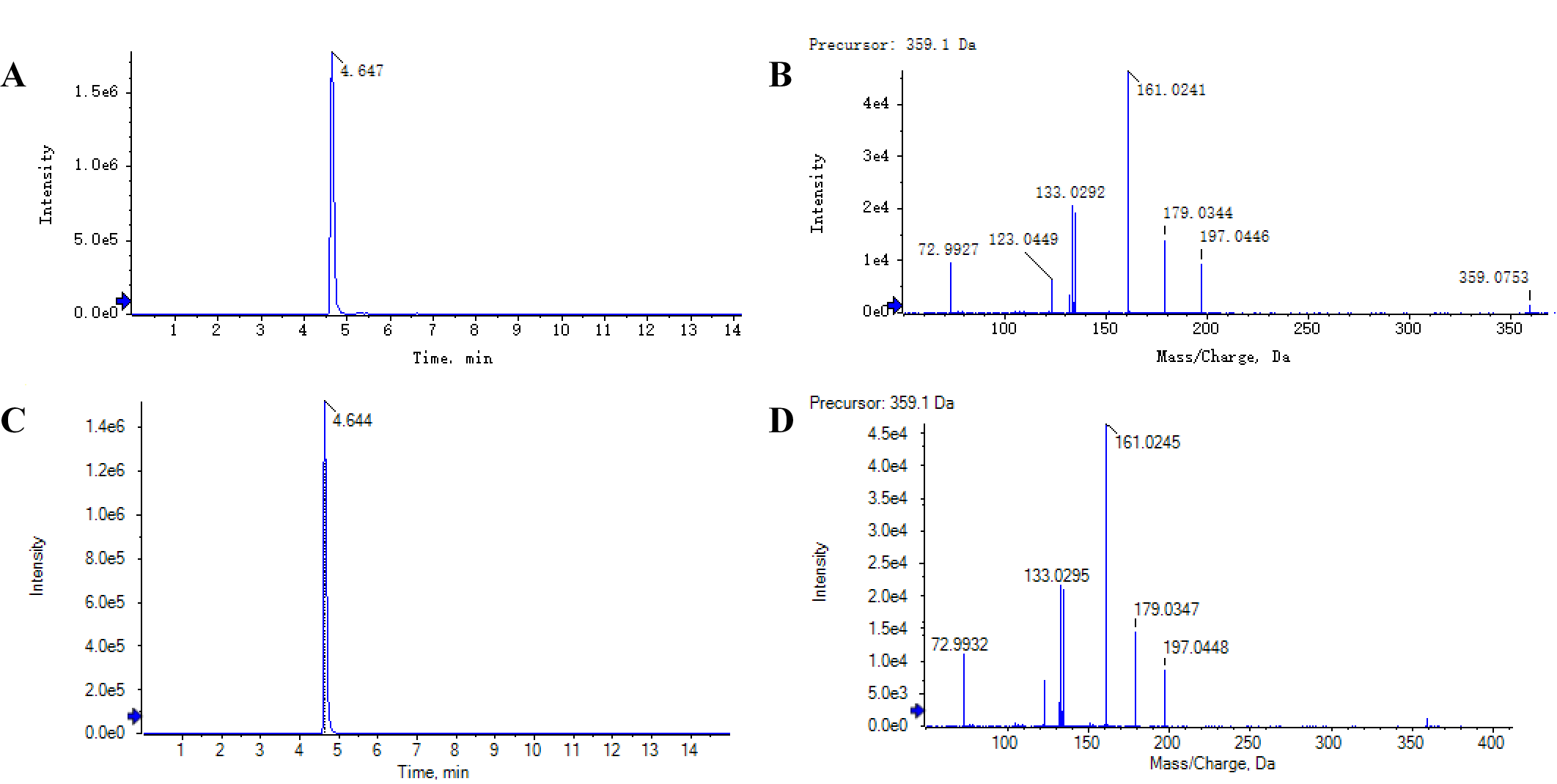


**Figure S2.** Qualitative analysis of rosmarinic acid in EEOS. (A) Precursor ion information for rosmarinic acid standard; (B) Product ion information for rosmarinic acid standard; (C) Precursor ion information of rosmarinic acid in EEOS extracts; (D) Product ion information of rosmarinic acid in EEOS extracts.


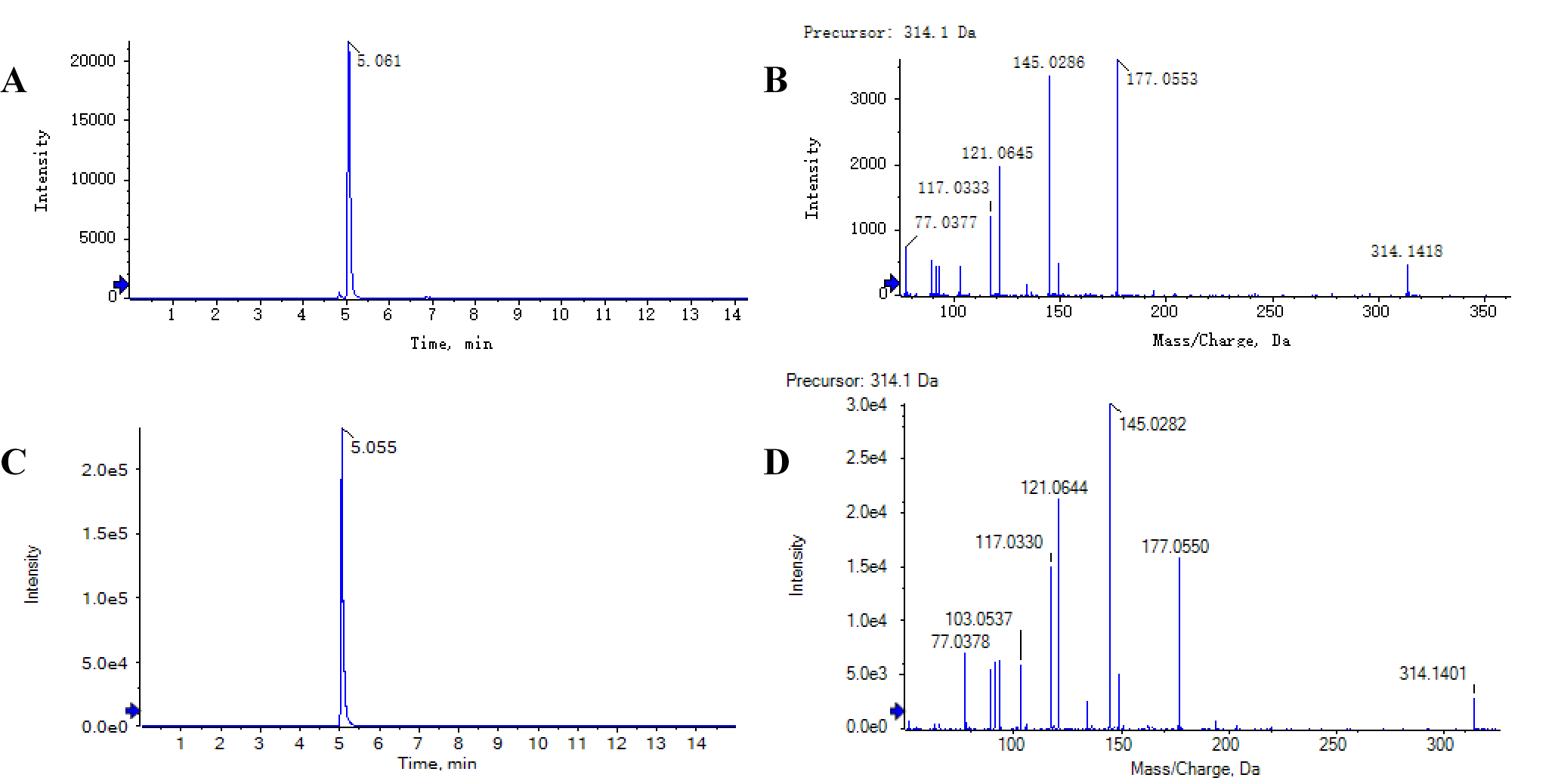


**Figure S3.** Qualitative analysis of N-trans-feruloyltyramine in EEOS. (A) Retention time (5.061 min) of N-trans-feruloyltyramine in EEOS extracts; (B) Product ions information of N-trans-feruloyltyramine in EEOS extracts; (C) Retention time (5.055 min) for N-trans-feruloyltyramine standard; (D) Product ions information for N-trans-feruloyltyramine standard.


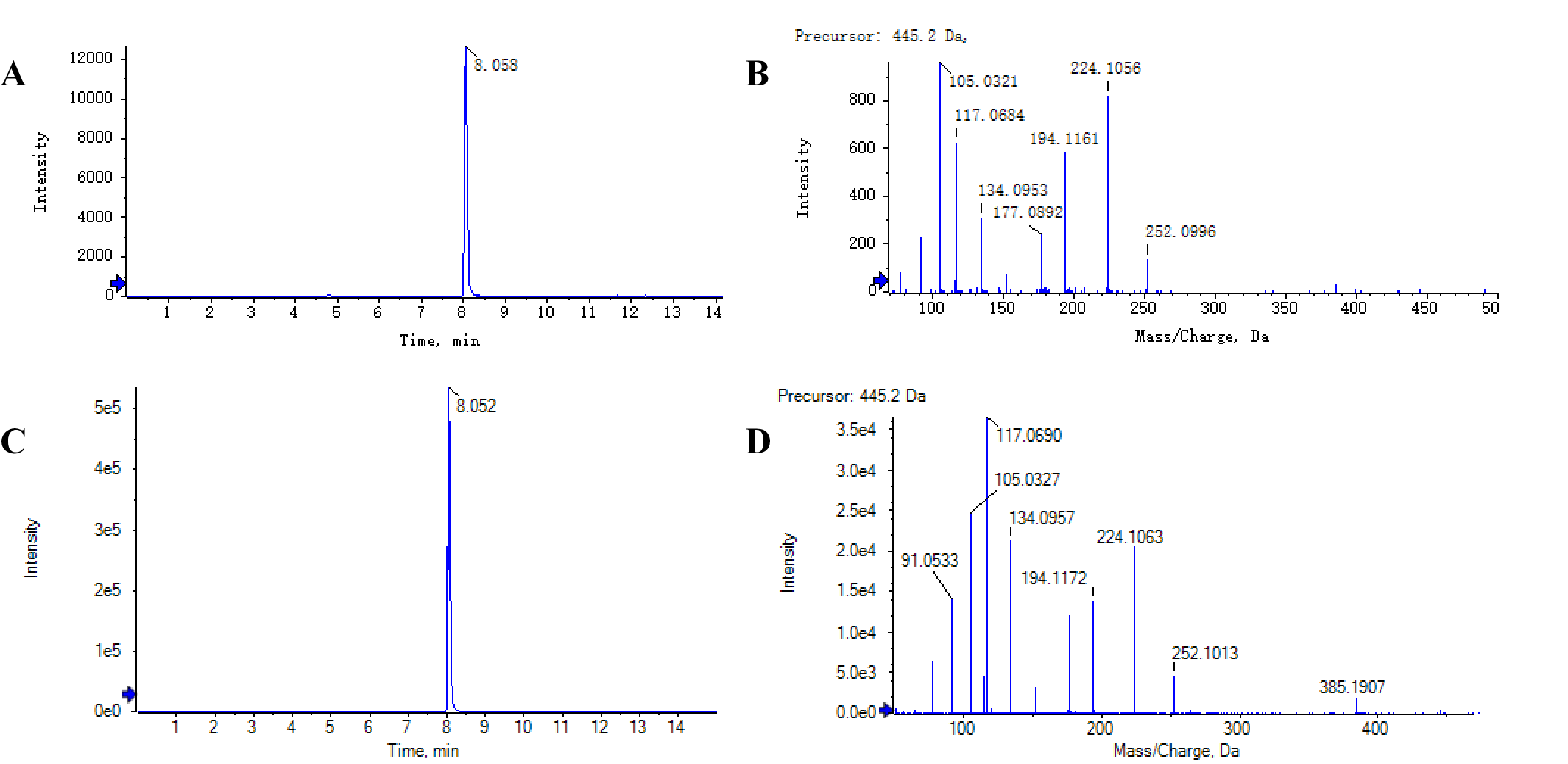


**Figure S4.** Qualitative analysis of asperglaucide in EEOS. (A) Retention time (8.058 min) of asperglaucide in EEOS extracts; (B) Product ions information of asperglaucide in EEOS extracts; (C) Retention time (8.052 min) for asperglaucide standard; (D) Product ions information for asperglaucide standard.


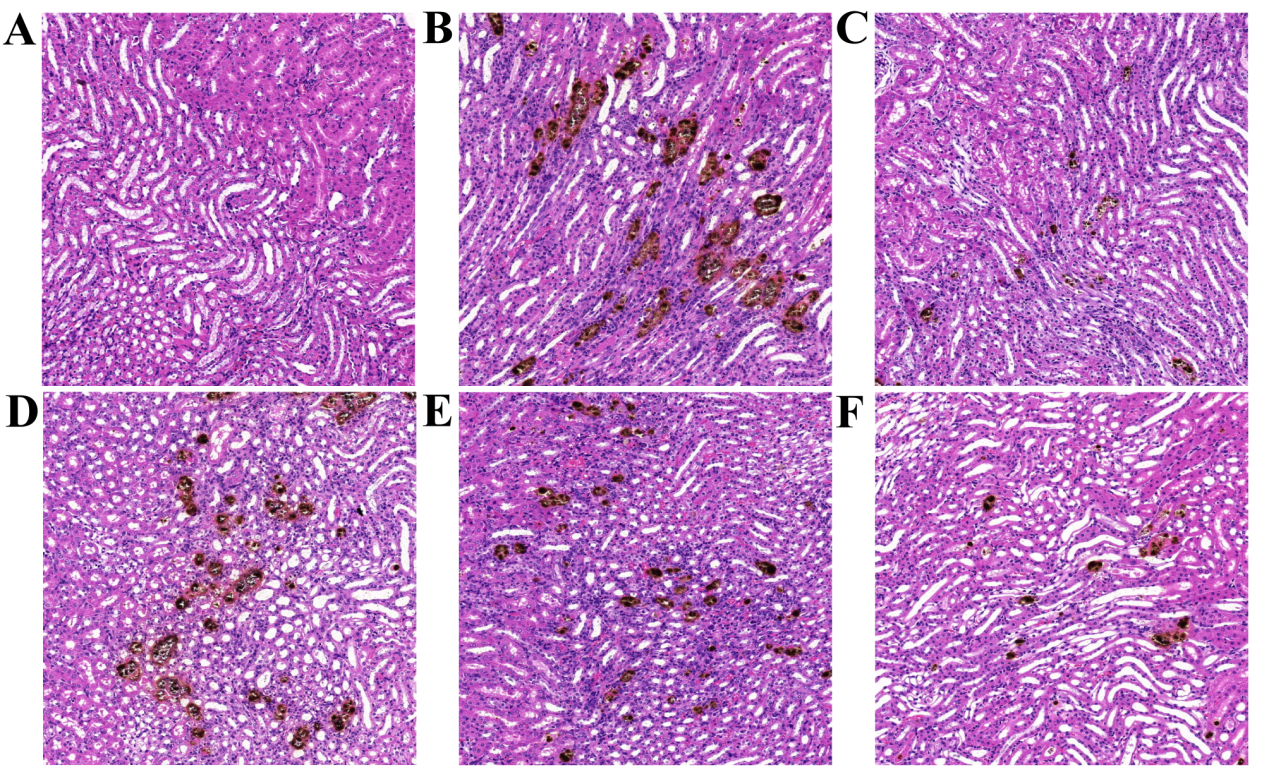


**Figure S5.** Representative photomicrographs of Von kossa stained renal sections(×400). (A) Control group showing no obvious crystalline deposition; (B) A large number of calcium deposition were observed in the model group kidney sections; (C) Calcium deposition was significantly in the LEEOS group; (D) Calcium deposition decreased slightly in the MEEOS group; (E) Calcium deposition obviously decreased in the HEEOS group; (F) Calcium deposition obviously decreased in the HEEOS group.


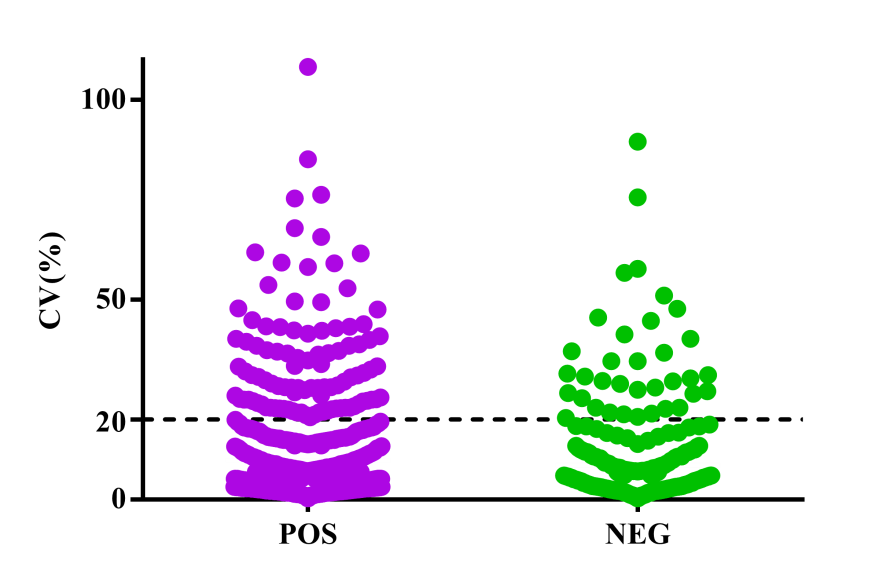


**Figure S6.** The coefficient of variation(CV) of QC samples. Ions with a CV value greater than 20% in the QC sample accounted for 11.61% and 10.57% of the total ions in the positive and negative modes.


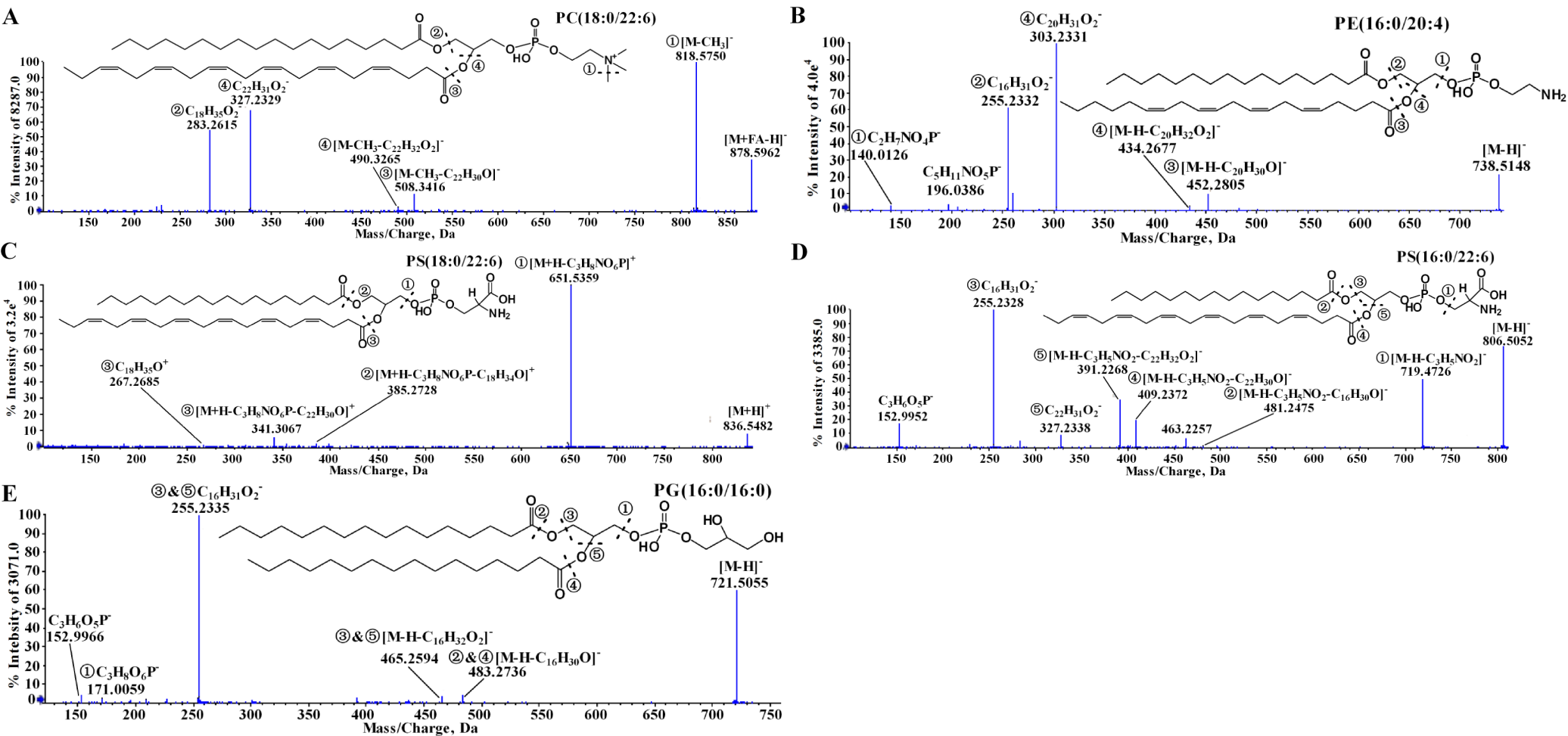


**Figure S7.** Identification of ions based on precursor ion and the corresponding product ions information. (A) Cracking information and structure analysis for PC(18:0/22:6); (B) Cracking information and structure analysis for PE(16:0/20:4); (C) Cracking information and structure analysis for PS(18:0/22:6); (D) Cracking information and structure analysis for PS(16:0/22:6); (E) Cracking information and structure analysis for PG(16:0/16:0).

**
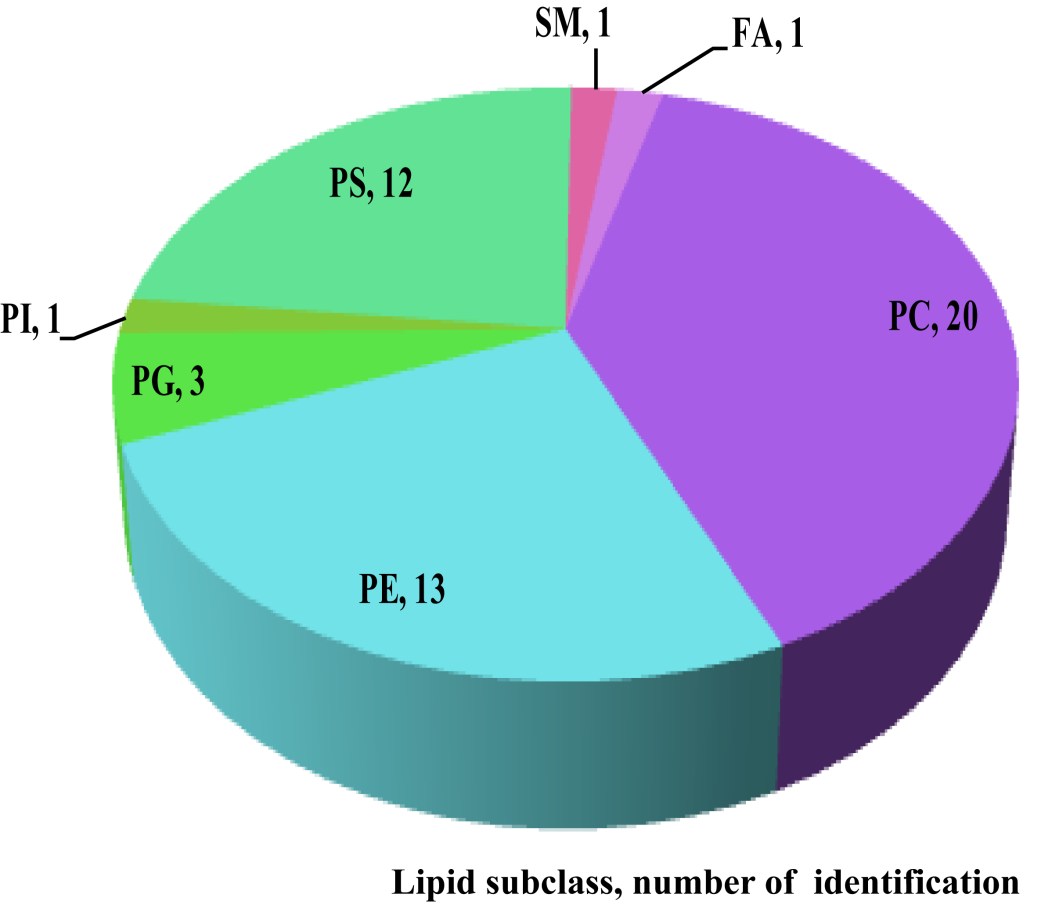
**

**Figure S8.** Summary of differential lipids color-coded by lipid subclass. 51 significant differential lipids from 7 lipid subclasses were identified between the model group and the EEOS group. PC: Glycerophosphocholine; PE: Glycerophosphoethanolamine; PG: Glycerophosphoglycerol; PI: Glycerophosphoinositol; PS: Glycerophosphoserine; SM: Sphingomyelin; FA: Fatty acyl.


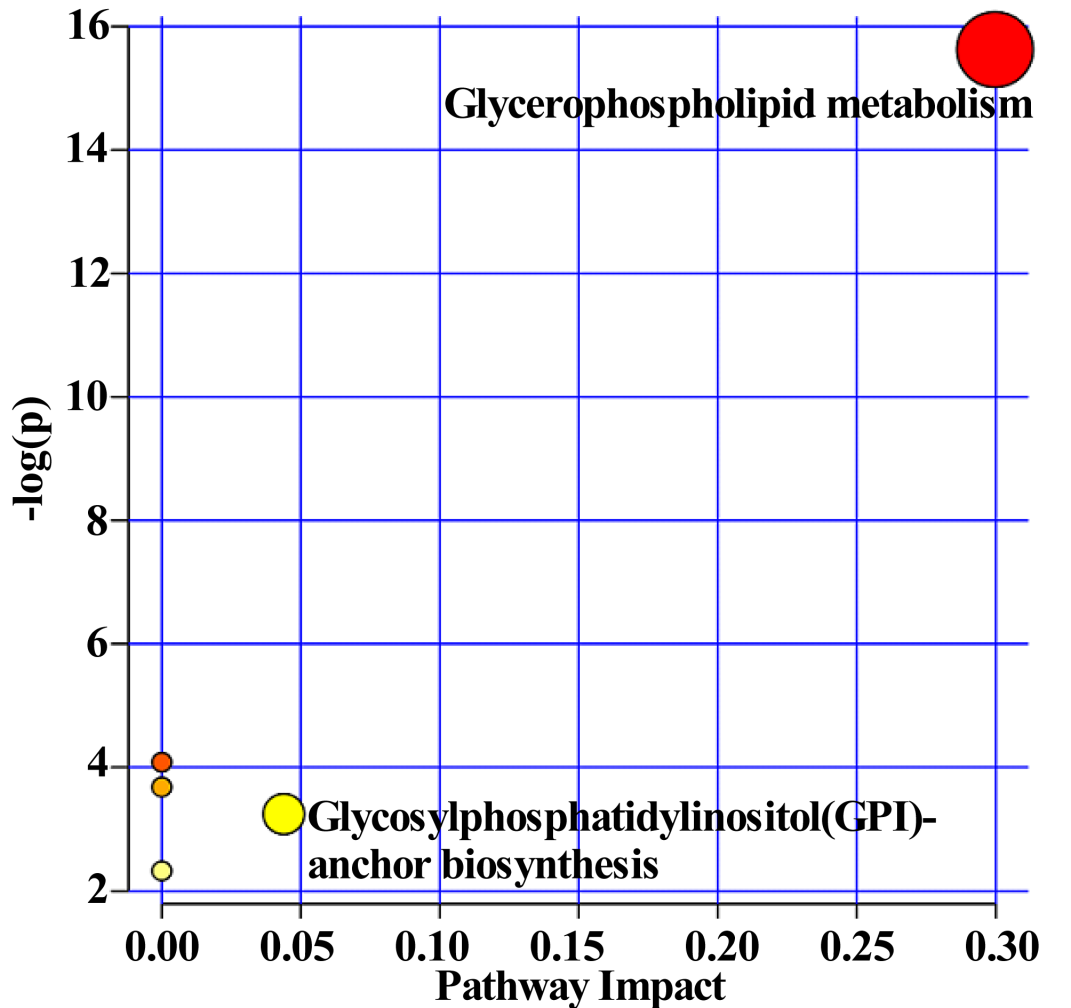


**Figure S9.** Summary of pathway impact analysis of differential lipids between EEOS group and model group. Glycerophospholipid metabolism pathway was significantly affected after administration of EEOS. The larger the circle is, the more lipids are focused, and the larger the -log (p) is, indicating that the significant difference between the metabolic pathway in the model group and the EEOS group was greater.

**Reference**

Akanae, W., Tsujihata, M., Yoshioka, I., Nonomura, N., and Okuyama, A. (2010). Orthosiphon grandiflorum has a protective effect in a calcium oxalate stone forming rat model. *Urological Research* 38**,** 89-96.

Yu-Sen, Z., Chen-Huan, Y., Hua-Zhong, Y., Zhi-Yuan, W., and Hua-Fang, C. (2012). Prophylactic effects of Orthosiphon stamineus Benth. extracts on experimental induction of calcium oxalate nephrolithiasis in rats. *Journal of Ethnopharmacology* 144**,** 761-767.
